# IL20RB promotes proliferation and migration in clear cell renal cell carcinoma and is associated with immune infiltration

**DOI:** 10.7717/peerj.20898

**Published:** 2026-03-10

**Authors:** Yufeng Liu, Yingmin Xie, Lingfei Yan, Yang Luo, Dawei Liu, Qing Li, Wu Xu, Tao Wang

**Affiliations:** 1 The Fifth Affiliated Hospital, Southern Medical University, Guangzhou, China; 2 Medical Research Institute, Guangdong Provincial People’s Hospital, Guangdong Academy of Medical Sciences, Southern Medical University, Guangzhou, China

**Keywords:** IL20RB, Prognosis, Tumor immune microenvironment, Clear cell renal cell carcinoma

## Abstract

**Background:**

IL20RB, interleukin 20 receptor subunit beta, functions as a cytokine receptor subunit coding gene and has been discovered to serve an essential function in human malignancies. However, the link between IL20RB expression, clinical outcomes, and tumor-infiltrating lymphocytes in clear cell renal cell carcinoma (ccRCC) remains unclear.

**Methods:**

The Cancer Genome Atlas (TCGA) was utilized to compile data on the IL20RB expression in both normal and ccRCC tissues. The link between IL20RB expression and clinicopathologic characteristics was examined utilizing the TCGA database. Kaplan-Meier survival curves were employed for performing the survival analysis. Furthermore, a protein network involving IL20RB was established using data from the GeneMANIA database. Gene Ontology (GO) and Kyoto Encyclopedia of Genes and Genomes (KEGG) were undertaken, and the relationship between IL20RB and tumor immune infiltration was examined via single-sample GSEA (ssGSEA). Additional examination of the link between tumor-infiltrating immune cells (TIIC) and IL20RB was executed utilizing the Tumor Immune Estimation Resource (TIMER) and TISIDB databases. IL20RB expression in tumor specimens was detected through immunohistochemistry (IHC). IL20RB expression levels in tumor cells were confirmed via Western blot analysis. Cell counting kit-8 (CCK-8) and colony formation assays evaluated IL20RB’s impact on ccRCC cell viability. Wound Healing and Transwell assays assessed IL20RB’s influence on ccRCC cell migration.

**Results:**

Peritumor samples exhibited notably reduced IL20RB expression compared to ccRCC samples. IL20RB expression levels correlated markedly with sample classification, lymph node status, tumor differentiation, and disease progression. Enhanced IL20RB expression is linked to poor Disease-Specific Survival (DSS) and Overall Survival (OS) in ccRCC patients (*p* < 0.01). Subsequently, a significant link was observed between IL20RB overexpression and immunomodulators, chemokines, and a heightened presence of infiltrating Treg, NK CD56 cells, Th1 cells, cytotoxic cells, and T helper cells in ccRCC. IHC showed that the IL20RB level in the adjacent normal tissues was notably diminished relative to that in ccRCC samples. IL20RB suppression through small interfering RNA (siRNA) markedly diminished ccRCC cell proliferation and migration.

**Conclusion:**

Heightened IL20RB expression is linked to a dismal prognosis and infiltration of immune cells in ccRCC, indicating its potential importance in the development of immunotherapeutic strategies.

## Introduction

Renal cell carcinoma (RCC) is the most deadly urological malignancy in terms of the yearly death rate, and its prevalence has been on the rise worldwide (Capitanio et al., 2019). As a heterogeneous malignancy, it is dominated by clear cell renal cell carcinoma (ccRCC), accounting for about 75–80% of renal cell carcinoma (Nabi et al., 2018). The majority of ccRCCs exhibit early inactivation of the tumor suppressor gene von Hippel-Lindau (VHL) (Frew & Moch, 2015; Ricketts & Linehan, 2018). Despite targeted therapy being one of the most commonly used conventional therapies to treat ccRCC, almost all patients eventually experience deterioration of their condition because ccRCC cells are resistant to drug-induced apoptosis (Sánchez-Gastaldo et al., 2017). Ferroptosis induction, an innovative regulated cell death mechanism, has surfaced as a promising therapeutic strategy for managing ccRCC (Atkins & Tannir, 2018; Wettersten et al., 2017; Miess et al., 2018; Zou et al., 2019). Currently, existing therapies only show promise for a limited portion of ccRCC patients, making it imperative to find more effective therapeutic targets. Additionally, there exists a pressing requirement to establish new biomarkers facilitating earlier detection and superior prognostic assessment of ccRCC.

IL20RB, interleukin 20 receptor subunit beta, characteristically establishes a heterodimeric cytokine receptor through combination with IL20RA or IL22RA1. The IL20RA/IL20RB complex serves as a receptor for IL19, IL20, and IL24, whereas the IL22RA1/IL20RB combination functions as a receptor for IL20 and IL24 (Blumberg et al., 2001). IL20RB represents the shared component between these receptor variants. IL20RB primarily operates through interactions with its specific ligands; these interleukins are classified within the IL20 cytokine subfamily (Madouri et al., 2018; Kunz et al., 2006). While IL20RB was originally discovered in association with various non-cancerous conditions, including psoriasis (Chan et al., 2006), rheumatoid arthritis (Sabat et al., 2007), vitiligo (Kingo et al., 2010), ulcerative colitis (Fonseca-Camarillo et al., 2013), glaucoma (Wirtz & Keller, 2016), asthma (Gong et al., 2014), endometriosis (Shao et al., 2016), and chronic rhinosinusitis (Lai et al., 2019), along with infectious diseases (Kong et al., 2020), recent findings suggest IL20RB demonstrates significant implications in malignant disorders. Current research indicates IL20RB exhibits crucial functions in various human cancers, encompassing colorectal adenocarcinoma (Zhang, Liu & Xu, 2019), breast cancer (Omarini et al., 2018), and esophageal carcinoma (Ma et al., 2014), though its significance in ccRCC remains to be elucidated.

The primary objective of this investigation centered on determining the possible connection between IL20RB expression, clinical parameters, and overall survival (OS) among ccRCC patients through multiple databases. Also, we examined how IL20RB is correlated with immune-related cells using the Tumor Immune Estimation Resource (TIMER). Using TISIDB databases, we then probed the association of IL20RB expression with its corresponding gene markers. We also investigated the IL20RB-interacting protein network using GeneMANIA. In ccRCC, substantial associations emerged between IL20RB expression and immune cell infiltration, along with poor prognoses. The results indicate that IL20RB might function as a valuable therapeutic target for ccRCC immunotherapy, marking a novel discovery in this field.

## Methodologies and materials

### Acquisition of transcriptomic and clinical datasets

RNA sequencing profiles together with matched clinicopathological annotations for ccRCC were downloaded from The Cancer Genome Atlas (TCGA; https://portal.gdc.cancer.gov/). Expression values of IL20RB were extracted and stratified into high- and low-expression groups based on the median level. Corresponding clinical characteristics were integrated for further analyses. As the TCGA database is publicly accessible, no additional ethical approval was required.

### Association with clinical characteristics

To investigate potential links between IL20RB expression and clinical-pathological variables, expression data were compared across pathological T, N, and M stages as well as histological grades. Statistical testing was carried out using the Kruskal–Wallis method implemented in R (stats v4.2.1 and car v3.1-0 packages). Graphical representations, including boxplots, were generated using the ggplot2 package (v3.4.4). Benjamini–Hochberg procedure was used to control the false discovery rate.

### Prognostic significance and construction of predictive models

Overall survival (OS), progression-free interval (PFI), and disease-specific survival (DSS) were analyzed in relation to IL20RB expression using Kaplan–Meier curves and Cox regression models. Analyses were performed with the “Clinical Significance” module of the Xiantao Academic platform (https://www.xiantao.love/). Patients were separated into high- and low-expression groups using the median IL20RB value. Associations with clinicopathological factors were evaluated by Wilcoxon signed-rank tests and logistic regression. Multivariate Cox models were applied to identify independent prognostic indicators. Based on these results, nomograms predicting 1-, 3-, and 5-year survival probabilities were established, with calibration plots used to evaluate predictive performance.

### Functional enrichment and interaction network analysis

Gene Ontology (GO) enrichment analyses were conducted with the clusterProfiler package (R v3.6.3), covering biological processes, molecular functions, and cellular components. Enrichment thresholds were set at a minimum count >3, enrichment factor >1.5, and *P* < 0.01. To further explore potential molecular interactions of IL20RB, a protein–protein interaction (PPI) network was generated using the GeneMANIA platform (http://www.genemania.org) (Zuberi et al., 2013), which integrates diverse genomic and proteomic datasets to predict functional associations among genes.

### Immune infiltration estimation by ssGSEA

Immune cell infiltration in ccRCC samples was assessed by applying the single-sample gene set enrichment analysis (ssGSEA) algorithm. Marker genes for 24 immune cell populations were obtained from Bindea et al. (2013). The abundance of each immune cell type was compared between IL20RB high- and low-expression groups, and associations were quantified using Spearman correlation coefficients. Analyses and visualization were implemented through the immune infiltration module of the Xiantao Academic platform.

### Pan-cancer immune infiltration analysis with TIMER2.0

To complement ccRCC-specific results, the Tumor Immune Estimation Resource (TIMER2.0; https://timer.cistrome.org/) (Li et al., 2020) was employed for a pan-cancer overview of IL20RB-related immune infiltration. TIMER2.0 encompasses 10,897 TCGA samples across 32 tumor types, enabling systematic evaluation of immune cell distributions as well as estimation of tumor purity.

### Immune regulatory landscape explored *via* TISIDB

The TISIDB platform (http://cis.hku.hk/TISIDB) (Ru et al., 2019), an integrative repository for tumor–immune system interactions, was employed to further investigate the immunological context of IL20RB in ccRCC. This resource combines data from high-throughput sequencing, literature mining, and immunological studies to provide a comprehensive overview of immune modulators. We specifically examined the correlations of IL20RB expression with tumor-infiltrating lymphocytes (TILs), immune stimulators, immune inhibitors, chemokines, and their corresponding receptors. These analyses were performed to assess the potential role of IL20RB in modulating the tumor immune microenvironment and predicting responses to immunotherapy.

### Immunohistochemistry

Immunohistochemical staining was performed as previously described (Xu et al., 2025), with slight modifications. Briefly, tumor and adjacent normal tissues were obtained from 12 patients who underwent radical nephrectomy for clear cell renal cell carcinoma at the Fifth Affiliated Hospital, Southern Medical University. Written informed consent was obtained from all patients, and the study was approved by the institutional Ethics Committee (Approval Number: 2023-MNWK-K-002). Formalin-fixed, paraffin-embedded tissues were sectioned, deparaffinized in xylene, and rehydrated through graded ethanol. Antigen retrieval was carried out in citrate buffer (pH 6.0; Biosharp, Beijing, China). Endogenous peroxidase activity was quenched with 3% H_2_O_2_, and nonspecific binding was blocked using 5% BSA (Biosharp, Beijing, China). The sections were then incubated overnight at 4 °C with an anti-IL20RB antibody (1:100; HUABIO, Hangzhou, China), followed by incubation with a secondary antibody (Dako, Glostrup, Denmark) for 1 h at room temperature. Immunoreactivity was visualized using DAB (Dako), counterstained with hematoxylin, dehydrated, and mounted. Staining intensity and distribution were quantified using ImageJ software. The H-score was calculated as: H-score = ∑(pi×i) = (% weak×1 + % moderate×2 + % strong×3).

### Cell culture and transfection

The HK-2, A498, Caki-1, and 786-O cell lines were procured from the American Type Culture Collection (ATCC). For HK-2 cells, cultivation occurred in DMEM/F-12 medium (Gibco, Waltham, MA, USA). The A498 cells underwent cultivation in MEM medium (Procell, Wuhan, China). For Caki-1 and 786-O cells, cultivation took place in RPMI 1640 medium (Gibco, Waltham, MA, USA). Each medium contained supplements of 10% fetal bovine serum (HyCyte, Suzhou, China) and 1% penicillin/streptomycin (Invitrogen, Waltham, MA, USA). The cells underwent incubation at 37 °C in an environment containing 5% CO_2_. All cell lines used in this study were routinely monitored for mycoplasma contamination using a commercial mycoplasma detection kit (Vazyme, Nanjing, China), and were confirmed to be mycoplasma-free. Cell transfection was performed utilizing Rfect V2 siRNA reagent (BIOG, Changzhou, China) per the supplier’s protocol. The design and synthesis of IL-20RB siRNAs were conducted by Beijing Tsingke Biotech Co., Ltd. The following sequences were utilized:

**Table:** 

IL-20RB siRNA1: 5′-GGUCCUGAGUGUGAUGUCAdTdT-3′
IL-20RB siRNA2: 5′-GGAUGGAGAUCACCAAAGAdTdT-3′
IL-20RB siRNA3: 5′-GGAGAAACAGUGUACUAUUdTdT-3′.

### Western blot

Cell specimens underwent lysis on ice utilizing RIPA buffer (Beyotime, Shanghai, China) comprising PMSF and protease inhibitor for a minimum of 10 min, accompanied by periodic agitation. Following centrifugation at 12,000 × g for 15 min at 4 °C, the liquid fraction was obtained. Protein concentration determination was performed using the BCA Protein Assay Kit (GLPBIO, Montclair, CA, USA) and standardized accordingly. The specimens underwent separation *via* 10% SDS-PAGE followed by transfer onto 0.45 µm PVDF membrane. Membrane blocking occurred at ambient temperature using rapid blocking buffer (Epigentek, Farmingdale, NY, USA), followed by overnight exposure to primary antibodies (IL20RB, ER63751, HUABIO, 1:1000; ACTIN, 66009-1-Ig, 1:50000; Proteintech, Madison, WI, USA) at 4 °C. Subsequently, the membrane experienced 1 × TBST washing and incubation with corresponding secondary antibody for 1.5 h at ambient temperature. Protein detection was accomplished using the Ultra High Sensitivity ECL Kit (GLPBIO, Montclair, CA, USA), with visualization through the Vilber Imaging System, utilizing ACTIN as the loading control.

### qRT-PCR

Quantitative real-time PCR (qRT-PCR) was performed to determine the mRNA expression levels of IL20RB in HK-2, A498, Caki-1, and 786-O cell lines. Total RNA was isolated using the EZ-press RNA Purification Kit (EZBioscience, Houston, TX, USA). RNA concentration and purity were evaluated with a Nanophotometer N50 ultra-micro spectrophotometer (IMPLEN, München, Germany). Complementary DNA (cDNA) was synthesized from total RNA using the Hifair^®^ III 1st Strand cDNA Synthesis SuperMix for qPCR (Yeasen Biotechnology, Shanghai, China). Subsequently, qRT-PCR analysis was conducted using ChamQ SYBR qPCR Master Mix (Vazyme Biotech Co., Ltd., Nanjing, China) on a Fluorescent Quantitative PCR Detection System (BIOER Technology, Hangzhou, China). GAPDH served as the endogenous control, and relative gene expression levels were calculated using the 2^−^ΔΔCt method. Primer sequences (Sangon Biotech, Shanghai, China) were as follows: IL20RB forward primer (ACTGAAGGTCCTGAGTGTGATGTC), reverse primer (TGAGGTCTGTGAGCCCAATGTG);

GAPDH: forward primer (GGTGTGAACCATGAGAAGTATGA), reverse primer (GAGTCC TTCCACGATACCAAAG).

### Wound healing

786-O cells were placed into 6-well plates with pre-drawn lines on the bottom. Transfection of IL20RB siRNA1 was performed following the instructions of the Rfect V2 siRNA reagent (BIOG, Changzhou, China). When cells reached 80%–90% confluence, scratch wounds were made using 200 µL pipette tips. After removing detached cells by washing, low-serum culture medium was added. Scratch areas were photographed at 0 h and 24 h under a microscope. ImageJ was used to measure scratch areas, and cell migration rate was calculated as: migration rate = (initial scratch area–current scratch area)/initial scratch area ×100%.

### Transwell assay

Post-siRNA transfection, 786-O cells underwent trypsinization, suspension in serum-free medium, enumeration, and concentration adjustment. A volume of 200 µL cell suspension was transferred to the Transwell insert’s upper chamber, while 600 µL of medium containing 10% FBS was introduced to the lower chamber. The cells were incubated for 24 h, after which the medium was removed, and the cells underwent fixation with 4% paraformaldehyde followed by 0.1% crystal violet staining. Cells that failed to migrate through the upper surface were carefully eliminated using a swab, and microscopic images were captured. For invasion analysis, PBS was utilized to dilute Matrigel at a 1:8 ratio. The upper chamber received 100 µL diluted Matrigel, followed by 1 h solidification at 37 °C. The subsequent steps were the same as the migration assay.

### CCK8

After transfection, the cells underwent digestion, resuspension, and concentration adjustment to 2 ×10^4^ cells/mL. A volume of 100 µL cell suspension was distributed into individual wells of a 96-well plate (six replicates per group). At 24-hour intervals, 10 µL of CCK-8 solution was introduced to each well. Following incubation for 1 h, absorbance values were ascertained utilizing a microplate reader.

### Colony formation assay

Post-intervention, cells were placed at 1,000 cells/well in 35 mm culture dishes. Culture medium was refreshed every 3 days. When clones grew sufficiently large and numerous, culture was terminated. Cells were fixed, stained, and imaged for result preservation.

### Statistical analysis

Data are denoted as the mean ± SD. Statistical analysis between two groups was executed utilizing *t*-test and Wilcoxon rank-sum test. The difference between several groups was compared with the Kruskal-Wallis test. For differential expression, we used an adjusted *p*-value (FDR) threshold of <0.05 and ∣log2FC∣>1. Enrichment thresholds were set at a minimum count >3, enrichment factor >1.5, and *P* < 0.01. Values of *P* < 0.05 indicated statistical significance.

## Results

### Tumor samples have higher levels of IL20RB expression than normal tissues

**Figure 1:**
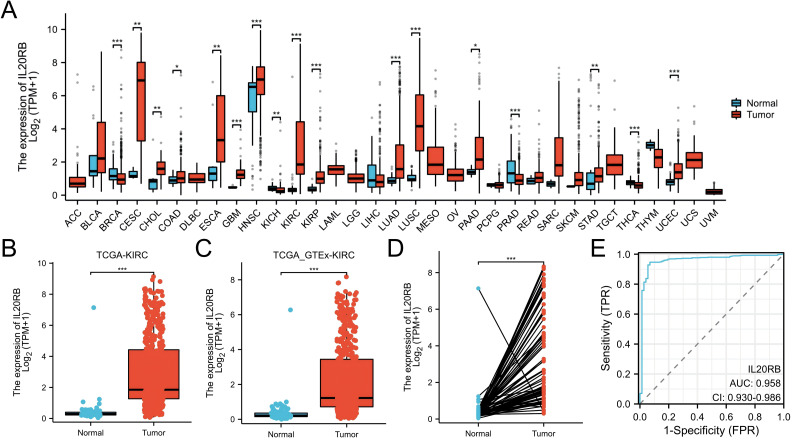
The expression profle of IL20RB in ccRCC. (A) Comparison of IL20RB expression in various human cancer tissues with that in normal samples; (B) Elevated IL20RB level was detected in KIRC tissues *versus* normal tissues; (C) Difference in IL20RB levels in KIRC and adjoining normal samples from the GTEx; (D) KIRC tissues showed higher levels of IL20RB expression compared to the corresponding normal tissues (*n* = 72); (E) Analyzing the ROC curve of IL20RB in patients with KIRC. (*p < 0.05, **p < 0.01, ***p < 0.001).

To determine whether IL20RB’s high expression is widespread in cancer, we first compared IL20RB expression in pan-cancers to adjacent normal tissues in the TCGA dataset (Fig. 1A). Cancers such as cervical squamous cell carcinoma (CESC), esophageal carcinoma (ESCA), and lung squamous cell carcinoma (LUSC) display elevated levels of IL20RB expression, whereas prostate adenocarcinoma (PRAD) and breast invasive carcinoma (BRCA) produce low levels. Using the TCGA databases, we predicted the expression patterns of IL20RB mRNA in 539 samples of ccRCC and 72 samples of normal tissue. Compared with normal tissue, ccRCC samples expressed markedly higher levels of IL20RB mRNA (Fig. 1B, *P* < 0.001). Further, IL20RB expression was compared in GTEx samples with adjoining ccRCC tissues and samples, and it was found to be up-regulated in ccRCC samples (Fig. 1C, *P* < 0.001). When compared with adjacent matched samples, 72 ccRCC samples showed significant increases in IL20RB expression (Fig. 1D, *P* < 0.001). Moreover, a receiver operating characteristic (ROC) curve was generated to estimate the diagnostic value of IL20RB levels. AUC value for IL20RB levels was 0.958 (CI = 0.930–0.986), demonstrating a promising diagnostic prospect (Fig. 1E). We have incorporated and examined supplementary datasets from GSE53757 and GSE66271 to corroborate our findings derived from the TCGA dataset (Fig. S1).

### Clinical characteristics associated with IL20RB expression

**Figure 2:**
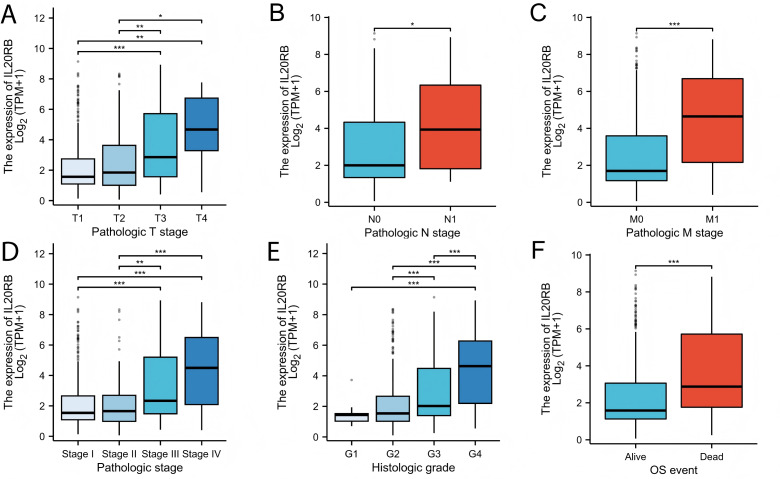
Clinical and pathological parameters of the KIRC correlated with IL20RB mRNA expression levels. (A) Pathologic T stage; (B) Pathologic N stage; (C) Pathologic M stage; (D) Pathologic stage; (E) Histologic grade; (F) OS event. G1, grade 1; G2, grade 2; G3, grade 3; G4, grade 4. (*p < 0.05, **p < 0.01, and ***p < 0.001).

We assessed whether IL20RB was correlated with different clinical-pathological features, which include pathologic T stage (T1,T2,T3,T4), pathologic N stage (N0 and N1), pathologic M stage(M0 and M1), histologic grade (grades 1, 2, 3, and 4), and pathologic stage (stages I, II, III, and IV). Higher pathologic T stage, N stage, M stage, were associated with higher IL20RB expression (Figs. 2A–2C, *p* < 0.05). Studies on cancer pathologic stages showed a dramatic upregulation of IL20RB in late-stage and middle cancers than in early cancers, suggesting that IL20RB might contribute to the development and migration of tumors (Fig. 2D). The study also showed a significant upregulation of IL20RB in the high-grade cancers in comparison to the low-grade cancers (Fig. 2E). Patients with dead event exhibited notably elevated levels of IL20RB compared to those with alive, event suggesting that IL20RB plays a role in malignancy (Fig. 2F).

Furthermore, consistent findings were obtained from the Fisher exact and chi-square tests (Table 1). Subsequently, the univariate logistic regression analysis demonstrated a substantial link between IL20RB level and various clinical variables, which include pathologic T stage (odds ratio [OR] = 3.048 [2.104–4.414], *P* < 0.001), Histologic grade (OR = 2.962 [2.081–4.216], *P* < 0.001), pathologic stage (OR = 3.502 [2.424–5.058], *P* < 0.001), and pathologic M stage (OR = 5.267 [2.909–9.535], *P* < 0.001) (Table 2). Nonetheless, no significant variations were observed in pathologic N stage (OR = 1.927 (0.650–5.713), *P* = 0.237), and gender (OR = 1.329 (0.931–1.896), *P* = 0.117) (Table 2). Based on these findings, IL20RB expression exhibited correlation with clinical characteristics.

**Table 1:** Link between IL20RB expression and clinicopathological characteristics in patients with KIRC.

Characteristics	Low expression of IL20RB	High expression of IL20RB	*P* value
n	270	271	
Pathologic T stage, n (%)			<0.001
T1	172 (31.8%)	107 (19.8%)	
T2	36 (6.7%)	35 (6.5%)	
T3	60 (11.1%)	120 (22.2%)	
T4	2 (0.4%)	9 (1.7%)	
Pathologic N stage, n (%)			0.230
N0	113 (43.8%)	129 (50%)	
N1	5 (1.9%)	11 (4.3%)	
Pathologic M stage, n (%)			<0.001
M0	237 (46.7%)	192 (37.8%)	
M1	15 (3%)	64 (12.6%)	
Pathologic stage, n (%)			<0.001
Stage I	172 (32%)	101 (18.8%)	
Stage II	33 (6.1%)	26 (4.8%)	
Stage III	48 (8.9%)	75 (13.9%)	
Stage IV	17 (3.2%)	66 (12.3%)	
Gender, n (%)			0.117
Female	102 (18.9%)	85 (15.7%)	
Male	168 (31.1%)	186 (34.4%)	
Age, n (%)			0.282
<= 60	128 (23.7%)	141 (26.1%)	
>60	142 (26.2%)	130 (24%)	
Histologic grade, n (%)			<0.001
G1	12 (2.3%)	2 (0.4%)	
G2	147 (27.6%)	89 (16.7%)	
G3	93 (17.4%)	114 (21.4%)	
G4	12 (2.3%)	64 (12%)	
Hemoglobin, n (%)			0.265
Low	119 (25.8%)	145 (31.5%)	
Normal	101 (21.9%)	91 (19.7%)	
Elevated	2 (0.4%)	3 (0.7%)	

**Table 2:** Logistic regression analysis of IL20RB expression.

Characteristics	Total (N)	OR (95% CI)	*P* value
Pathologic T stage (T3&T4 *vs.* T1&T2)	541	3.048 (2.104–4.414)	<0.001
Pathologic N stage (N1 *vs.* N0)	258	1.927 (0.650–5.713)	0.237
Pathologic M stage (M1 *vs.* M0)	508	5.267 (2.909–9.535)	<0.001
Gender (Male *vs.* Female)	541	1.329 (0.931–1.896)	0.117
Histologic grade (G3&G4 *vs.* G1&G2)	533	2.962 (2.081–4.216)	<0.001
Pathologic stage (Stage III&Stage IV *vs.* Stage I&Stage II)	538	3.502 (2.424–5.058)	<0.001

### Prognostic value of IL20RB expression in ccRCC

**Figure 3:**
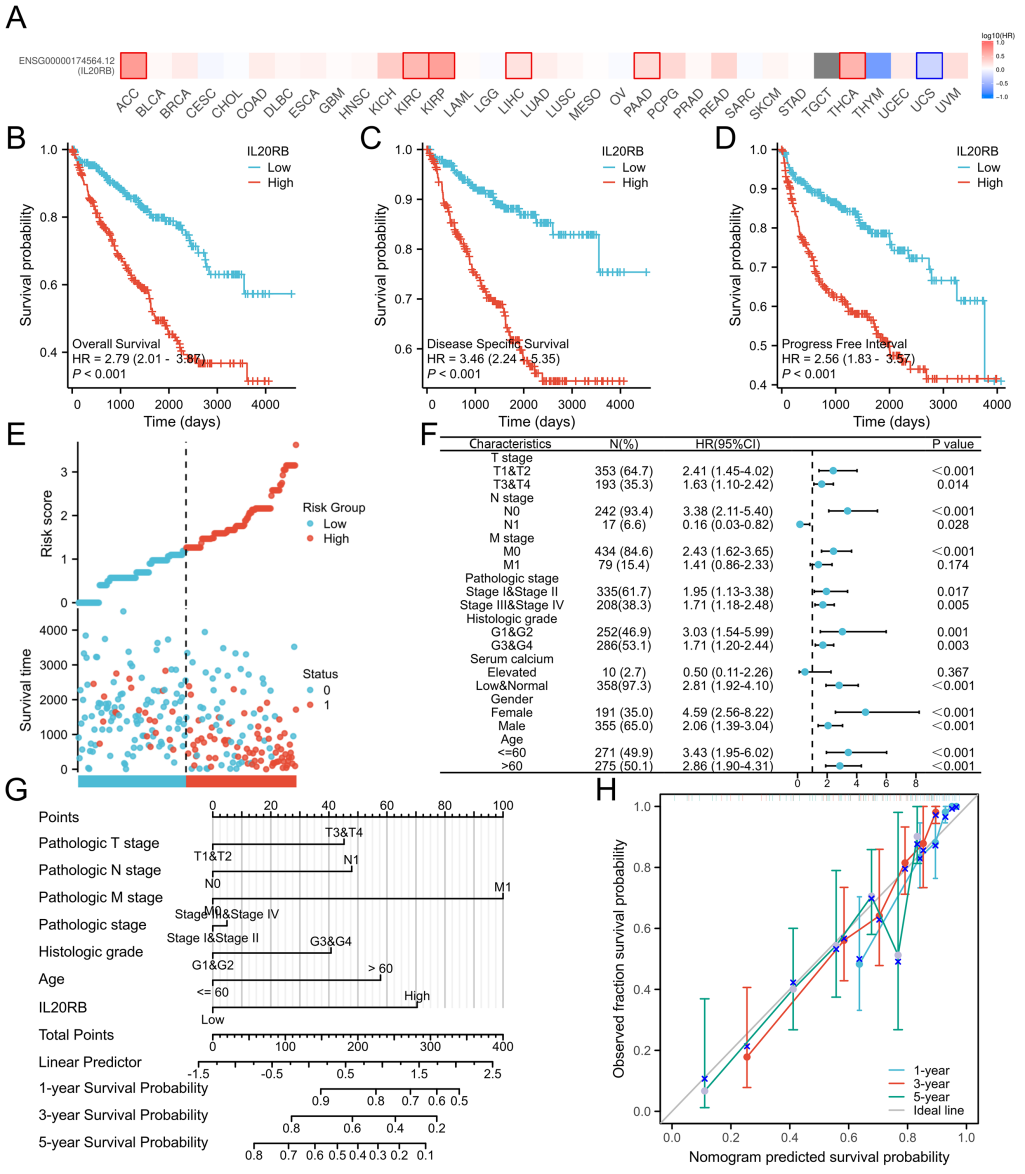
Expression of IL20RB as a prognostic indicator. (A) Establishment of an OS heatmap of the IL20RB gene. (B–D) Results indicated a significant IL20RB upregulation in patients with poor prognoses relative to those with high expression levels, as evidenced by OS, DSS, and PFI outcomes (*P* < 0.001). (E) Risk scores and survival status of IL20RB gene in KIRC patients. (F) Evaluation of the prognosis linked to IL20RB expression in clinical subgroups. (G) A nomogram was developed utilizing the clinical characteristics of IL20RB expression. (H) Multivariate Cox regression calibration chart displays the model’s predictive ability.

Subsequently, an OS heatmap of the IL20RB gene was generated (Fig. 3A). In a variety of tumors, such as adrenocortical carcinoma (ACC), kidney renal clear cell carcinoma (KIRC), kidney renal papillary cell carcinoma (KIRP), liver hepatocellular carcinoma (LIHC), pancreatic adenocarcinoma (PAAD), and thyroid carcinoma (THCA), high expression of IL20RB is markedly linked to poor prognosis. We explored the link between IL20RB expression rates and prognostic outcomes (OS, DSS, PFS) using TCGA database-derived data. IL20RB expression enhancement was shown to be markedly linked to worse OS, DSS, and PFS in the study population, with hazard ratios (HR) of 2.79 (95% CI [2.01–3.87]), 3.46 (95% CI [2.24–5.35]), and 2.56 (95% CI [1.83–3.57]), respectively, all with a *p*-value < 0.001 (Figs. 3B–3D). Analysis examining IL20RB expression within distinct subgroups revealed elevated IL20RB levels in T1-T2 stage (HR = 2.41 (1.45–4.02), *P* < 0.001), pathologic stage I–II (HR = 1.95 (1.13–3.38), *P* = 0.017), Histologic grade G1-G2 (HR = 3.03 (1.54–5.99), *P* = 0.001) (Fig. 3F). We developed a clinical prognostic risk score using the stages T, N, M, pathologic stage, histologic grade and IL20RB expression in ccRCC (Fig. 3G). The model’s predictive accuracy was evaluated through a calibration plot (Fig. 3H). According to the findings, IL20RB expression levels could better predict patient survival at 3- and 5-year intervals.

**Figure 4:**
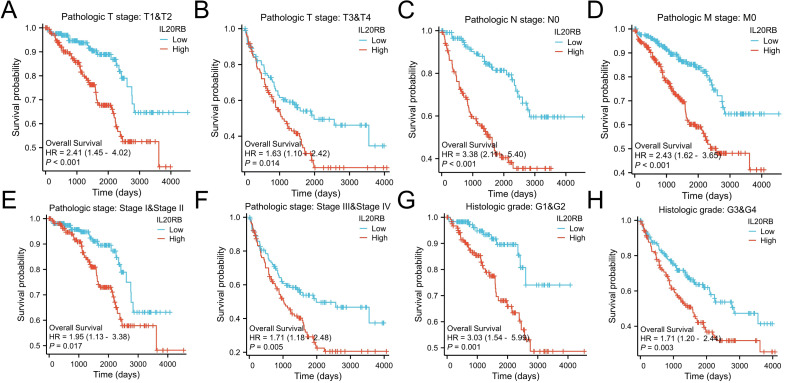
Survival curves for KIRC patients stratified by various clinical characteristics among those with high and low IL20RB expression. Kaplan–Meier survival for OS among groups stratified by (A) Pathologic T1&T2 stage, (B) Pathologic T3&T4 stage, (C) Pathologic N0 stage, (D) Pathologic M0 stage, (E) Pathologic stage I/II, (F) Pathologic stage III/IV, (G) Histologic grade 1& grade 2, (H) Histologic grade 3 & grade 4 in KIRC patients.

Additional evaluation of overall survival outcomes was performed among patients exhibiting distinct phenotypes in the low *versus* high IL20RB expression categories. Additionally, the examination revealed that elevated IL20RB expression corresponded to poorer OS outcomes in patients classified as T1/2 stage (*P* < 0.001), T3/4 stage (*p* = 0.014), N0 (*p* < 0.001), M0 (*P* < 0.001), and M1 (*p* = 0.029) (Fig. 4). All these results indicate that IL20RB expression levels are associated with ccRCC prognoses.

### GO/KEGG enrichment analysis related to IL20RB gene expression and IL20RB -interaction genes in ccRCC tissue

**Figure 5:**
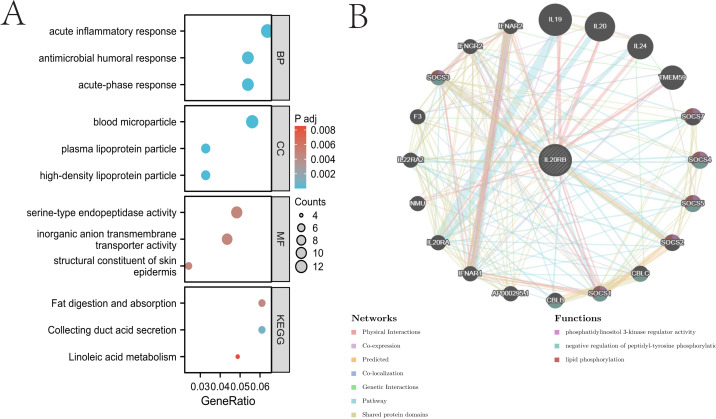
GO enrichment analysis related to IL20RB gene expression and IL20RB-interaction proteins in ccRCC. (A) GO enrichment analysis was conducted on differentially expressed genes identified through IL20RB expression screening. (B) Annotation of IL20RB-interacting proteins.

A gene expression profiling analysis was undertaken to understand the biological function of IL20RB in ccRCC. The analysis identified 3,805 downregulated and 171 upregulated genes that were substantially linked to IL20RB expression (Padj < 0.05 and logFC > 1). Additionally, analyses of gene ontology (GO) enrichment were carried out based on IL20RB expression levels. The biological processes of the IL20RB gene are primarily correlated with acute-phase response, acute inflammatory response, antimicrobial humoral response, protein-lipid complex remodeling and so forth (Table 3, Fig. 5A). The Genemania tool was utilized for analyzing the interaction network of IL20RB gene to identify its interplay in ccRCC advancement. Figure 5B lists the highest-ranked genes and their respective gene names, which include CCDC15, GYPC, HAVCR2, HLA-DMA, LNPEP, SCML2, TYROBP, SH2D4A, THOP1, TPP2.

### Correlation between immune cell infiltration and IL20RB expression levels

**Figure 6:**
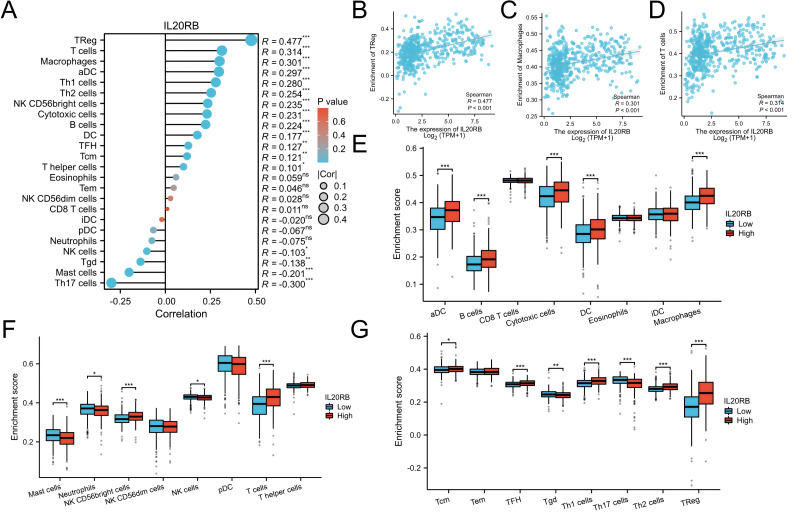
Analysis of the link between immune infiltration and IL20RB expression. (A) The association of the expression of IL20RB with immune cells. (B–D) Infiltration of specific immune cells is correlated with IL20RB expression. (E–G) A comparison of enrichment scores in the high-expression and low-expression subgroups of IL20RB. (ns) indicates *P* ≥ 0.05; * *P* < 0.05; ** *P* < 0.01; *** *P* < 0.001; **** *P* < 0.0001.

Following this, the correlation between IL20RB expression levels and 24 distinct immune cell types was assessed in clear cell renal cell carcinoma. IL20RB levels showed positive correlations with various immune components, notably regulatory T cells (Treg, *r* = 0.333), T lymphocytes (*r* = 0.314), and macrophages (*r* = 0.301), while demonstrating inverse relationships with Th17 cells (*r* =  − 0.300), all achieving statistical significance (*p* < 0.001) (Figs. 6A–6D). Further examination uncovered significant differences in IL20RB expression among diverse infiltrating immune cell populations, including aDC, B cells, cytotoxic cells, DC, mast cells, NK CD56 bright cells, TFH, Tgd, and TH1 cells (Figs. 6E–6G). Specifically, we incorporated data from CIBERSORT algorithm to examine correlations with a more comprehensive panel of 22 immune cell subtypes (Fig. S2). We also performed correlation analyses between IL20RB expression and immune checkpoint molecules (Fig. S3). These results indicate a critical function of IL20RB in mediating immune cell infiltration in ccRCC.

**Table 3:** GO enrichment analysis results.

Ontology	ID	Description	GeneRatio	BgRatio	*p* value	p.adjust
BP	GO:0006953	Acute-phase response	11/204	48/18,800	2.98e−12	6.05e−09
BP	GO:0002526	Acute inflammatory response	13/204	113/18,800	2.92e−10	2.97e−07
BP	GO:0019730	Antimicrobial humoral response	11/204	122/18,800	9.2e−08	6.23e−05
BP	GO:0034368	Protein-lipid complex remodeling	6/204	30/18,800	7.25e−07	0.0003
BP	GO:0034369	Plasma lipoprotein particle remodeling	6/204	30/18,800	7.25e−07	0.0003
CC	GO:0034364	High-density lipoprotein particle	7/214	27/19,594	1.24e−08	2.89e−06
CC	GO:0072562	Blood microparticle	12/214	147/19,594	7.79e−08	6.24e−06
CC	GO:0034358	Plasma lipoprotein particle	7/214	36/19,594	1.07e−07	6.24e−06
CC	GO:1990777	Lipoprotein particle	7/214	36/19,594	1.07e−07	6.24e−06
CC	GO:0032994	Protein-lipid complex	7/214	39/19,594	1.92e−07	8.95e−06
MF	GO:0004252	Serine-type endopeptidase activity	10/207	174/18,410	2.83e−05	0.0047
MF	GO:0015103	Inorganic anion transmembrane transporter activity	9/207	151/18,410	5.37e−05	0.0047
MF	GO:0030280	Structural constituent of skin epidermis	5/207	37/18,410	5.57e−05	0.0047
MF	GO:0008509	Anion transmembrane transporter activity	13/207	315/18,410	6.06e−05	0.0047
MF	GO:0008236	Serine-type peptidase activity	10/207	191/18,410	6.23e−05	0.0047
KEGG	hsa04966	Collecting duct acid secretion	5/82	27/8,164	6.14e−06	0.0008
KEGG	hsa04975	Fat digestion and absorption	5/82	43/8,164	6.45e−05	0.0045
KEGG	hsa00591	Linoleic acid metabolism	4/82	29/8,164	0.0002	0.0085
KEGG	hsa00590	Arachidonic acid metabolism	5/82	61/8,164	0.0003	0.0119
KEGG	hsa04721	Synaptic vesicle cycle	5/82	78/8,164	0.0011	0.0297

### IL20RB expression is correlated with immunoregulators in ccRCC

**Figure 7:**
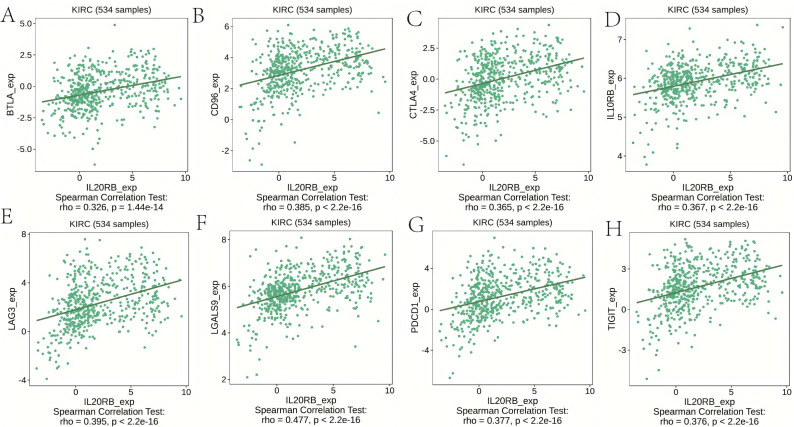
IL20RB expression is correlated with immunoinhibitors in KIRC. (A) BTLA (*r* = 0.326), (B) CD96 (*r* = 0.385), (C) CTLA4 (*r* = 0.365), (D) IL-10RB (*r* = 0.367), (E) LAG3 (*r* = 0.395), (F) LGALS9 (*r* = 0.477), (G) PDCD1 (*r* = 0.377), and (H) TIGIT (*r* = 0.376).

**Figure 8:**
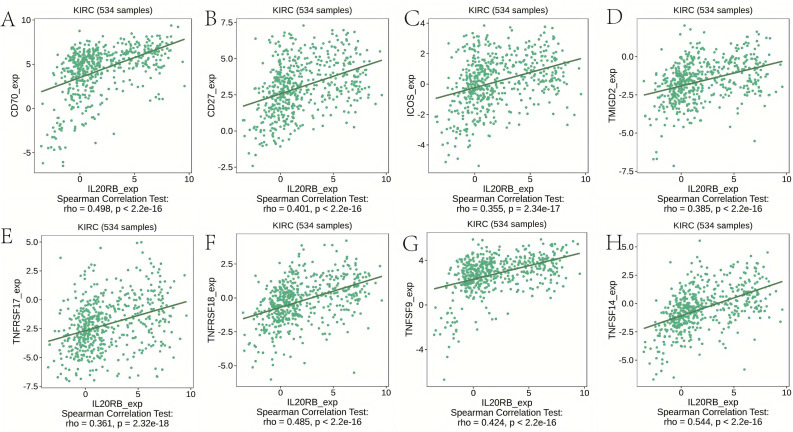
IL20RB expression is associated with immunostimulators in KIRC. (A) CD70 (*r* = 0.498), (B) CD27 (*r* = 0.401), (C) TMIGD2 (*r* = 0.385), (D) TNFRSF17 (*r* = 0.361), (E) TNFRSF18 (*r* = 0.485), (F) ICOS (*r* = 0.355), (G) TNFSF9 (*r* = 0.424), and (H) TNFSF14 (*r* = 0.544).

The expression of IL20RB exhibited significant correlations with immune inhibitors (all *p* < 2.2e–16), including BTLA (*r* = 0.326), CD96 (*r* = 0.385), CTLA4 (*r* = 0.365), IL-10RB (*r* = 0.367), LAG3 (*r* = 0.395), LGALS9 (*r* = 0.477), PDCD1 (*r* = 0.377), and TIGIT (*r* = 0.376) (Fig. 7). Additionally, the expression of IL20RB demonstrated close associations with immunostimulators (all *p* < 2.2e–16), including CD70 (*r* = 0.498), CD27 (*r* = 0.401), TMIGD2 (*r* = 0.385), TNFRSF17 (*r* = 0.361), TNFRSF18 (*r* = 0.485), ICOS (*r* = 0.355), TNFSF9 (*r* = 0.424), and TNFSF14 (*r* = 0.544) (Fig. 8). Based on the above findings, IL20RB performs a key function in controlling immune responses and could impact how tumors evade the immune system.

### IL20RB demonstrates elevated expression in ccRCC and promotes cancer cell growth, motility, and infiltration

**Figure 9:**
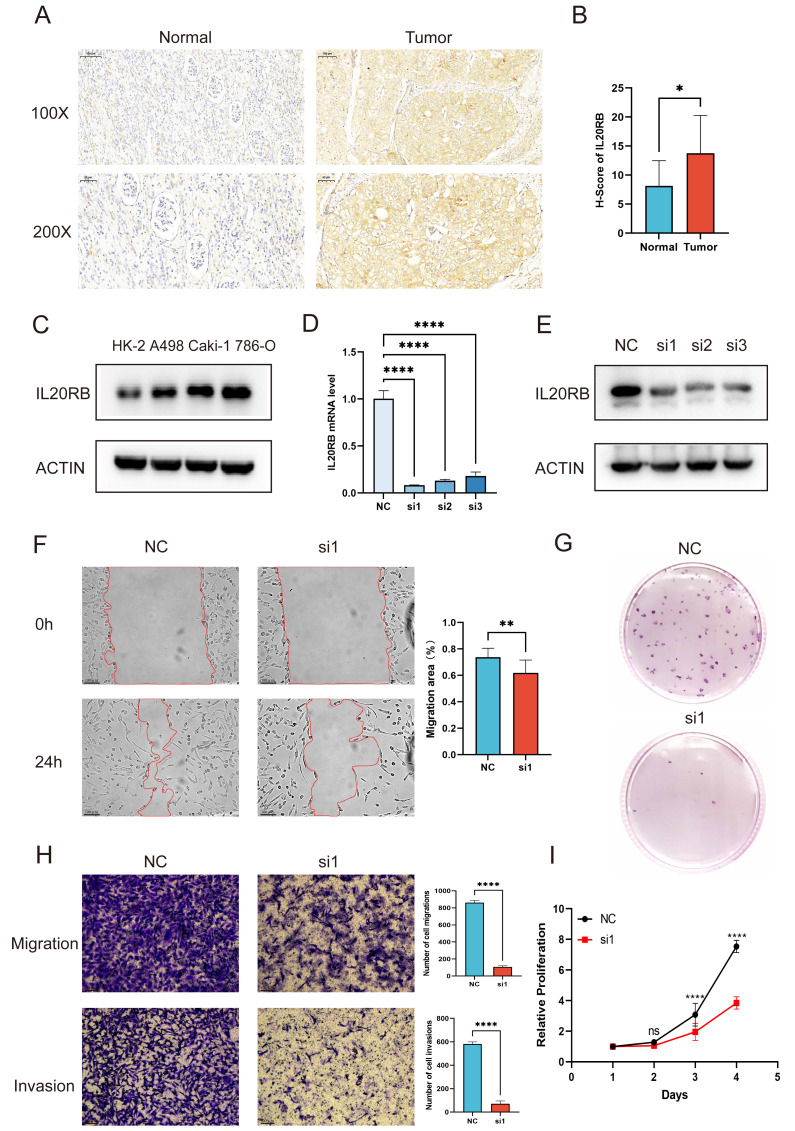
IL20RB is highly expressed in ccRCC and contributes to tumor proliferation, migration, and invasion. (A, B) IL20RB immunohistochemistry and statistical analysis in ccRCC tumor *versus* paired adjacent normal tissues (*n* = 12). Scale bars: 100 µm at 100× magnification and 50 µm at 200× magnification. (C) Western blot was utilized to evaluate IL20RB protein levels in the normal cell line HK-2 and the RCC cell lines A498, Caki-1, and 786-O. (D, E) The knockdown efficiency of IL20RB in 786-O cells was assessed by qRT-PCR and Western blot. (F, H) IL20RB knockdown impaired the migratory and invasive capacities of 786-O cells, as shown by Transwell and wound-healing assays. (G, I) IL20RB knockdown suppressed the proliferative potential of 786-O cells, as determined by colony-formation and CCK-8 assays. * *P* < 0.05; ** *P* < 0.01; *** *P* < 0.001; **** *P* < 0.0001.

To assess IL20RB levels in ccRCC, specimens from tumor and neighboring normal tissues were obtained from 12 individuals receiving radical nephrectomy for ccRCC at the medical center. Immunohistochemical analysis demonstrated elevated IL20RB presence in cancerous sections compared to adjacent healthy tissue (Figs. 9A–9B). Examining cellular models revealed heightened IL20RB quantities in malignant cell lines A498, Caki-1, and 786-O *versus* normal HK-2 cells, with 786-O exhibiting peak expression, making it suitable for subsequent investigations (Fig. 9C). After designing and synthesizing three siRNAs targeting IL20RB, all three siRNAs showed effective inhibitory effects, and the knockdown efficiency was validated by qRT-PCR and Western blot, with siRNA1 ultimately selected for further research (Figs. 9D–9E). Migration and invasion capabilities, assessed through wound healing and Transwell experiments, showed substantial reduction upon IL20RB suppression (Figs. 9F, 9H). Furthermore, CCK-8 and colony formation studies indicated that diminished IL20RB levels markedly decreased tumor cell growth (Figs. 9G, 9I).

## Discussion

ccRCC is characterized by substantial molecular heterogeneity, which has prompted increasing interest in identifying biomarkers relevant to disease aggressiveness and therapeutic response. In this context, we investigated the expression profile of IL20RB using transcriptomic data derived from TCGA. Comparative analyses revealed that IL20RB expression was markedly dysregulated in multiple tumor types, with a pronounced upregulation observed in KIRC relative to normal renal tissue. Notably, elevated IL20RB expression in ccRCC was preferentially enriched in tumors exhibiting aggressive clinicopathological characteristics, including advanced local invasion, higher histological grade, and metastatic involvement. Consistent with these observations, survival stratification analyses demonstrated that patients with high IL20RB expression experienced significantly inferior OS, PFI, and DSS. Together, these findings support a potential role for IL20RB in ccRCC malignancy and underscore its relevance to disease progression rather than merely reflecting expression heterogeneity.

The present study identified IL20RB as a novel, independent prognostic biomarker for ccRCC. Elevated expression of IL20RB was significantly associated with improved OS, DSS, and PFS in ccRCC patients. This favorable prognostic relationship may be attributed to its inverse correlation with advanced disease features, including higher T stage, N stage, M stage, and poorer histologic grade. To facilitate personalized clinical assessment, we integrated IL20RB expression levels with key clinical parameters (TNM stage and histologic grade) to construct and validate multivariate prognostic nomograms. These models provide a refined tool for predicting individual patient outcomes, potentially aiding in risk stratification and therapeutic decision-making.

In this investigation, IL20RB emerged as an independent prognostic indicator for ccRCC; furthermore, the IL20 subfamily, encompassing IL19, IL20, and IL24, participates in heightened inflammatory responses and anti-inflammatory processes, including tissue protection and renewal (Logsdon et al., 2012; Rutz, Wang & Ouyang, 2014; Foster et al., 2004; Cui, Cui & Leng, 2019). IL19 exhibits direct effects on immune cells, IL20 demonstrates substantial impact on skin inflammation, and IL24 induces apoptosis across various cancer types (Ouyang et al., 2011; Blumberg et al., 2001; Lebedeva et al., 2002). IL20RB, functioning as a component of the IL20 subfamily receptor, participates in inflammatory processes and malignant conditions. Our analysis further demonstrated a significant correlation between IL20RB expression and the extent of immune cell infiltration in KIRC. Immune cell infiltration is a fundamental component of tumor biology and plays a critical role in shaping the tumor microenvironment, thereby modulating therapeutic responsiveness to chemotherapy, immunotherapy, and radiotherapy and ultimately influencing clinical outcomes (Curigliano, 2018; Klauschen et al., 2018). The analysis revealed substantial positive correlations between IL20RB expression and Treg (*r* = 0.477), macrophages (*r* = 0.301), and T cells (*r* = 0.314), while displaying notable negative correlations with Th17 cells (*r* =  − 0.300), Mast cells (*r* =  − 0.201), and Tgd cells (*r* =  − 0.138), all demonstrating *p*-values <0.01. Mounting evidence indicates that immune cell infiltration correlates closely with RCC patient prognosis (Braun et al., 2020). This investigation demonstrated that Tregs and macrophages exhibited elevated expression in patients with high IL20RB levels. Research has established that Tregs and Macrophages serve crucial functions in cancer progression and metastasis (Haebe et al., 2021; Xia et al., 2020). While preliminary studies suggest that IL20RB may play a role in modulating immune responses, the current evidence remains limited and requires further validation. The potential therapeutic applications of targeting IL20RB, such as in autoimmune diseases or cancer, are supported by *in vitro* and animal models, but clinical translation remains uncertain due to the complexity of its signaling mechanisms and potential off-target effects. Future research should focus on elucidating the precise molecular interactions of IL20RB in human pathophysiology, as well as conducting rigorous clinical trials to assess its safety and efficacy.

Through the GeneMANIA database analysis, proteins demonstrating potential interactions with IL20RB were identified, particularly SCML2 and TYROBP, which hold key positions in the protein-protein interaction network. Research has established SCML2 as a significant modulator of chemotherapy resistance, exhibiting elevated expression across multiple tumor types (Peng et al., 2023). Studies have revealed elevated TYROBP as an indicator predicting unfavorable outcomes in clear cell renal cell carcinoma (Lv et al., 2022). Furthermore, investigations into the potential downstream mechanisms underlying IL20RB’s oncogenic function in KIRC utilized GO and KEGG enrichment analyses. The GO enrichment analysis indicated IL20RB’s connection to acute-phase response and serine-type endopeptidase activity, while KEGG analysis revealed enrichment in pathways including collecting duct acid secretion and fat digestion and absorption.

Through additional analyses of 12 pairs of ccRCC patient paracancerous and tumor tissues collected, the upregulation of IL20RB in ccRCC was further confirmed in an independent dataset. In addition, further experiments were performed to assess the importance of IL20RB for ccRCC cells by knocking down the expression of IL20RB in ccRCC cells. In subsequent CCK-8 assays, cloning assays, it was observed that knockdown of IL20RB was sufficient to inhibit ccRCC cell proliferation, our group also supplemented migration and invasion experiments to demonstrate that IL20RB expression regulates ccRCC cell migration and invasion ability. Taken together, our database analysis and related experimental results are sufficient to demonstrate that IL20RB expression promotes ccRCC proliferation, migration, and invasive ability.

Despite uncovering the possible impact of IL20RB on the immune infiltration and prognosis in KIRC, our study is still constrained by various limitations. Several limitations of the present study should be acknowledged. First, the majority of the data were derived from publicly available online databases, which are periodically updated and may introduce variability into the analytical results. Second, although the clinical and immunological relevance of IL20RB in ccRCC was suggested by our analyses, its functional role and underlying immunoregulatory mechanisms have not yet been validated using *in vivo* models. Future studies incorporating well-designed animal experiments will be necessary to further substantiate these findings.

## Conclusion

Our findings highlight that IL20RB is prominently expressed in KIRC tissues, and its upregulation is strongly linked to worse survival outcomes in KIRC. Additionally, IL20RB expression is linked to the regulation of T-cell exhaustion, macrophages, and Treg cells. The results suggest that IL20RB may influence the recruitment and activity of immune cells, such as regulatory T cells (Tregs), which are known to suppress antitumor immune responses. Additionally, IL20RB expression has been correlated with poor clinical outcomes in certain malignancies, suggesting its utility as a prognostic biomarker (Jiang et al., 2024). Future studies should also evaluate the potential of IL20RB as a biomarker for predicting response to immunotherapy, such as immune checkpoint inhibitors. In summary, IL20RB represents a promising yet underexplored target in cancer immunology.

##  Supplemental Information

10.7717/peerj.20898/supp-1 Supplemental Information 1 Raw Data for WB

10.7717/peerj.20898/supp-2 Supplemental Information 2 Raw Data for colony formation

10.7717/peerj.20898/supp-3 Supplemental Information 3 Raw Data for invasion

10.7717/peerj.20898/supp-4 Supplemental Information 4 Raw Data migration

10.7717/peerj.20898/supp-5 Supplemental Information 5 Raw Data for scratch assay

10.7717/peerj.20898/supp-6 Supplemental Information 6 Raw Data for CCK8

10.7717/peerj.20898/supp-7 Supplemental Information 7 Original data

10.7717/peerj.20898/supp-8 Supplemental Information 8 Raw numerical data for Figures 9B, 9E, and 9G (H-score, and cell migration)

10.7717/peerj.20898/supp-9 Supplemental Information 9 Supplementary datasets from GEO

10.7717/peerj.20898/supp-10 Supplemental Information 10 Association of immune infiltration with IL20RB

10.7717/peerj.20898/supp-11 Supplemental Information 11 Correlation between IL20RB expression and immune checkpoint molecules

10.7717/peerj.20898/supp-12 Supplemental Information 12 Cell line authentication

10.7717/peerj.20898/supp-13 Supplemental Information 13 Original data for PCR

10.7717/peerj.20898/supp-14 Supplemental Information 14 Original data for WB

## Additional Information and Declarations

### Data Availability

The following information was supplied regarding data availability: The code and scripts for the bioinformatic analyses is available in ZenodoWANG, T. (2025). R code for analysis [Data set]. Zenodo. https://doi.org/10.5281/zenodo.17105697. The raw data are available in the Supplemental Files.
