# Supplementary figures and images for: IL20RB promotes proliferation and migration in clear cell renal cell carcinoma and is associated with immune infiltration
Source: PeerJ. 2026 Mar 10;14:e20898. doi: 10.7717/peerj.20898 (PMC12985013; doi:10.7717/peerj.20898)

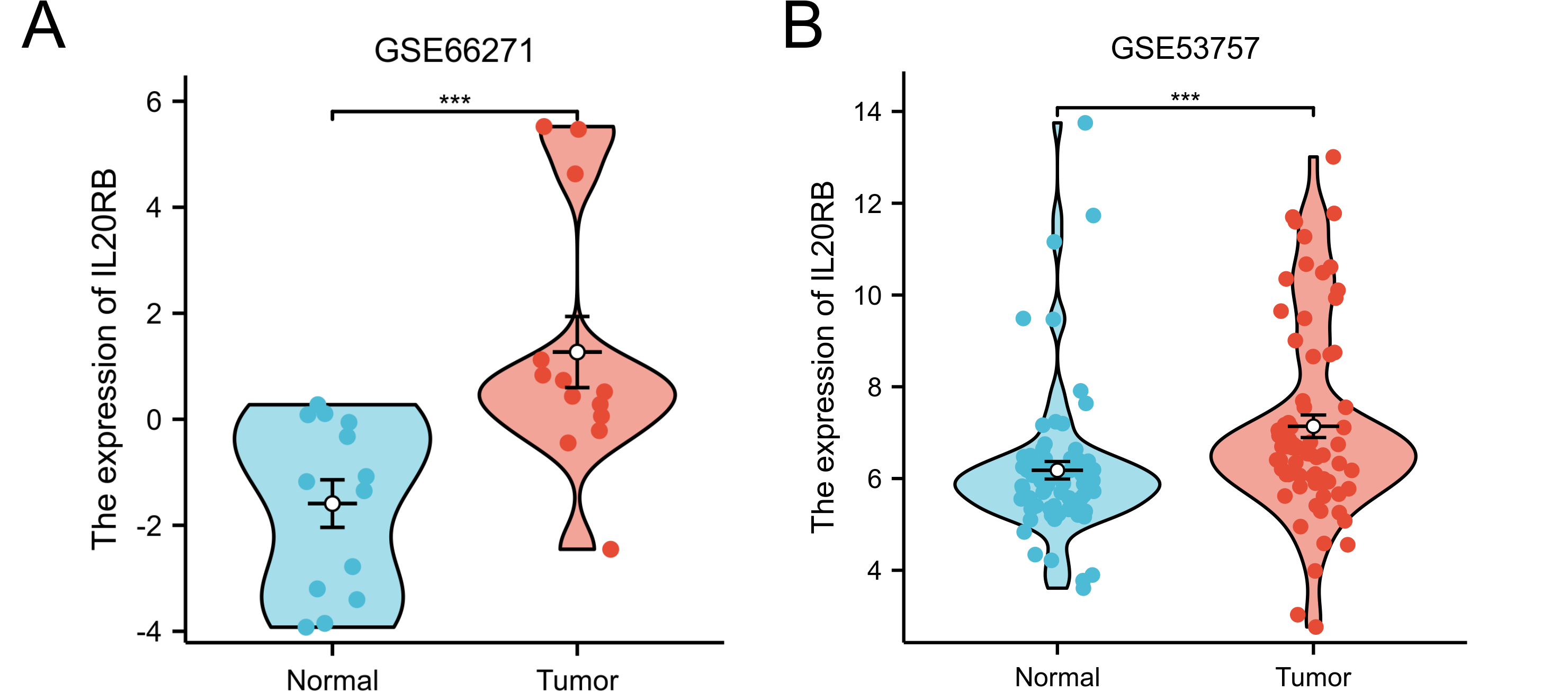

Supplement: Supplemental Information 9 [file peerj-14-20898-s009.tiff]

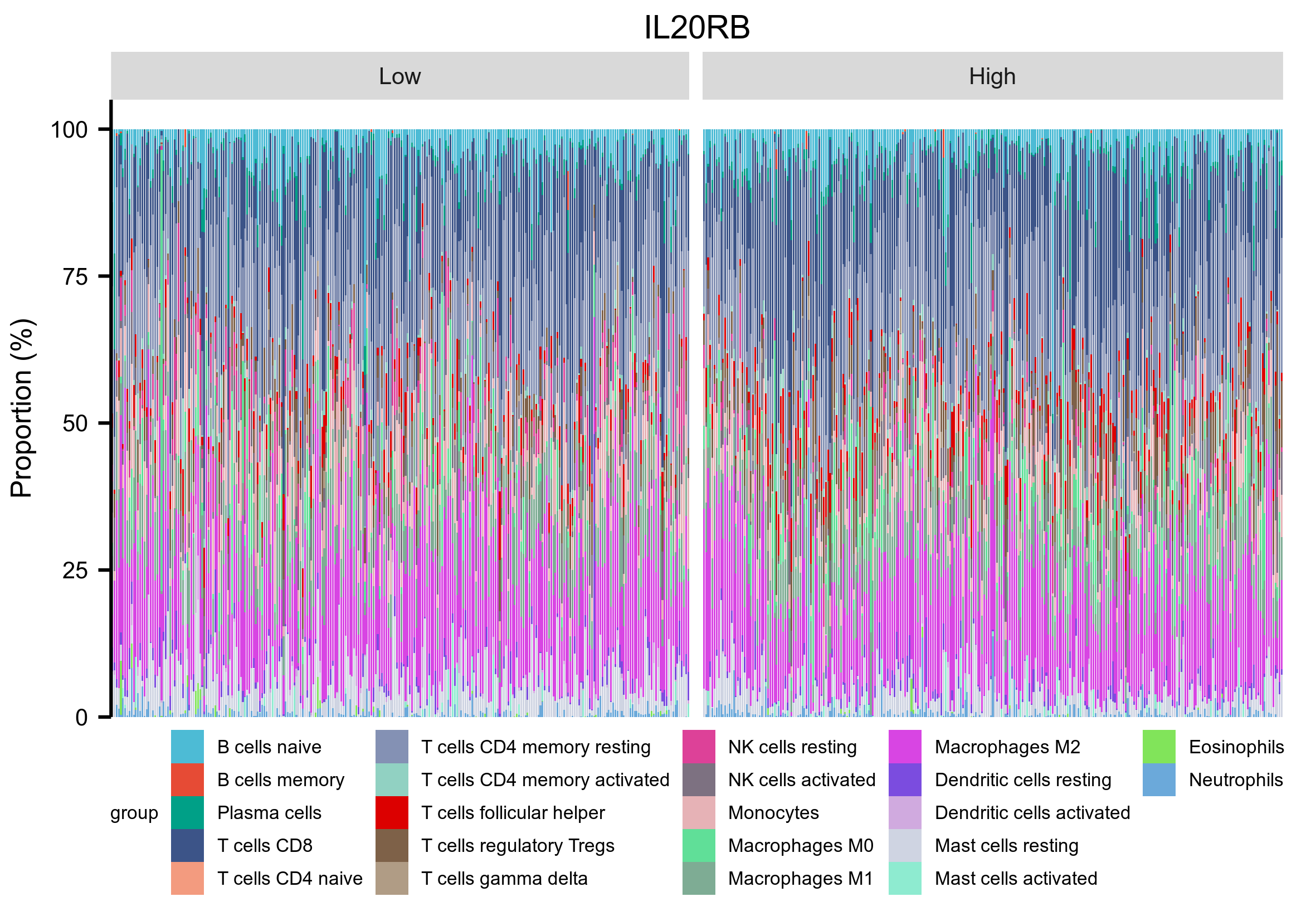

Supplement: Supplemental Information 10 [file peerj-14-20898-s010.tiff]

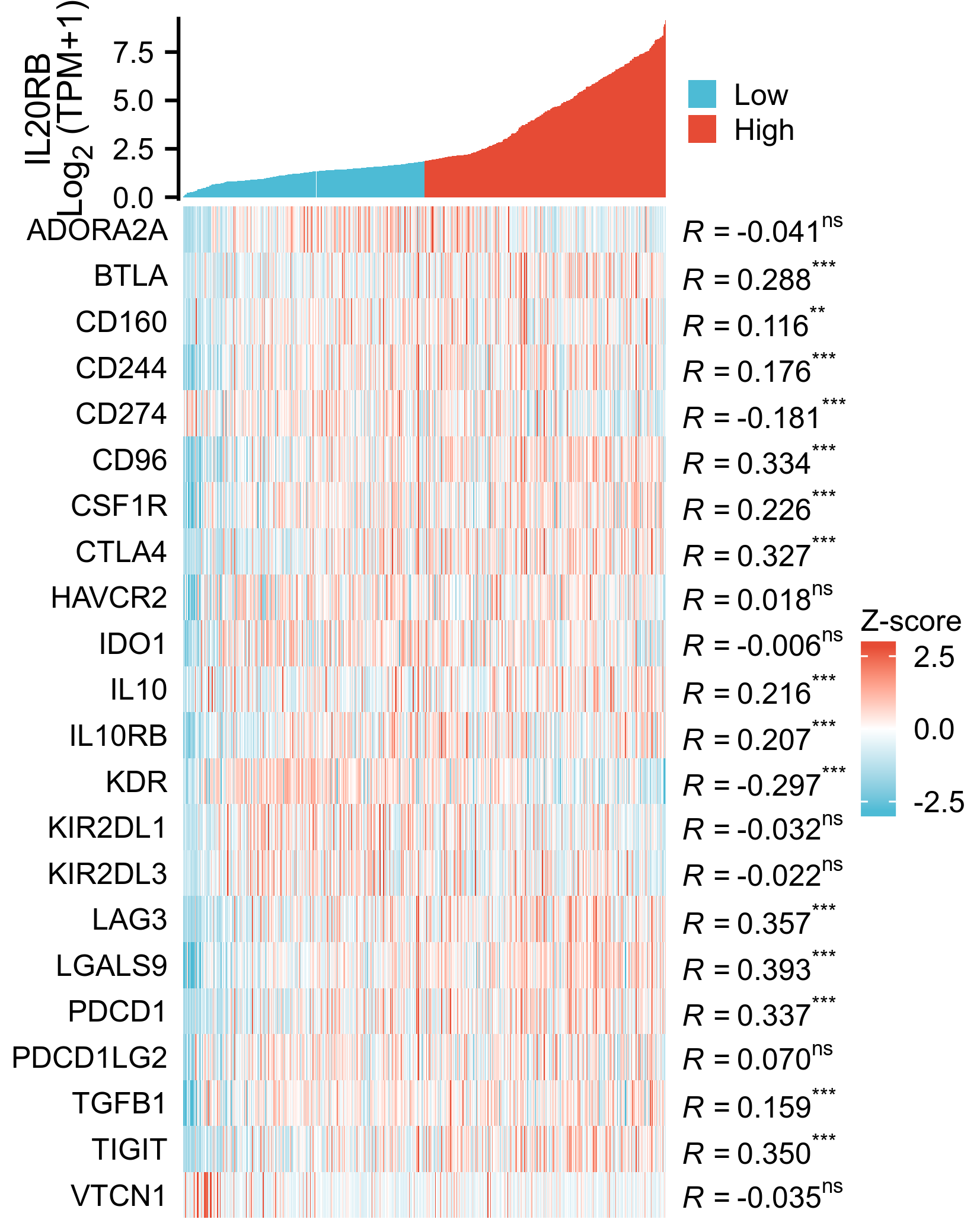

Supplement: Supplemental Information 11 [file peerj-14-20898-s011.tiff]
